# Health behavior profiles and association with mental health status among US active-duty service members

**DOI:** 10.3389/fpubh.2024.1324663

**Published:** 2024-02-22

**Authors:** Bolanle Olapeju, Zoé Mistrale Hendrickson, Patrice Shanahan, Omar Mushtaq, Anwar E. Ahmed

**Affiliations:** ^1^Department of Preventive Medicine and Biostatistics, Uniformed Services University of the Health Sciences, Bethesda, MD, United States; ^2^Department of Health, Behavior and Society, Johns Hopkins Bloomberg School of Public Health, Baltimore, MD, United States; ^3^Department of Behavioral and Community Health Sciences, University of Pittsburgh School of Public Health, Pittsburgh, PA, United States; ^4^Department of Medical and Clinical Psychology, Uniformed Services University of the Health Sciences, Bethesda, MD, United States

**Keywords:** behavior, latent class, profiles, risk, servicemembers, United States

## Abstract

**Introduction:**

This study investigated the clustering of health behaviors among US active duty servicemembers (ADSM) into risk profiles and explored the association between these profiles with ADSM sociodemographic characteristics and mental health status.

**Methods:**

This study utilized secondary data from the 2018 Health Related Behaviors Survey (HRBS), a Department of Defense (DoD) self-administered online survey. Health behaviors included physical activity, screen use, sleep habits, tobacco/substance use, alcohol drinking, preventive health care seeking and condom use at last sex/having multiple sexual partners. Past-year mental health status was measured using the Kessler Screening Scale for Psychological Distress (K6). Latent class analysis (LCA) on health behaviors was used to cluster ADSMs into risk profiles. Multivariable logistic model was used to examine whether ADSM characteristics and mental health status were associated with ADSMs' risk profiles.

**Results:**

The LCA identified a four-class model that clustered ADSMs into the following sub-groups: (1) Risk Inclined (14.4%), (2) High Screen Users (51.1%), (3) Poor Sleepers (23.9%) and (4) Risk Averse (10.6). Over a tenth (16.4%) of ADSMs were categorized as having serious psychological distress. Being male, younger, less educated, in the Army, Marine Corps or Navy were associated with higher odds of being Risk Inclined (AOR ranging from 1.26 to 2.42). Compared to the reference group of Risk Adverse ADSMs, those categorized as Risk Inclined (AOR: 8.30; 95% CI: 5.16–13.36), High Screen Users (AOR: 2.44; 95% CI: 1.56–3.82) and Poor Sleepers (AOR: 5.26; 95% CI: 3.38–8.19) had significantly higher odds of having serious psychological distress.

**Discussion:**

Study findings suggest opportunities to tailor behavioral and health promotion interventions for each of the distinct risk profiles. For example, ADSM described as Risk Inclined may benefit from preventive mental health services. Solutions for ADSM described as Poor Sleepers may include education on sleep hygiene; instituting duty schedules; and shifting military cultural norms to promote sleep hygiene as a pathway to optimal performance and thus military readiness. ADSM with low-risk behavior profiles such as those described as Risk Averse may prove beneficial in the roll-out of interventions as they act as peer-educators or mentors.

## 1 Introduction

The link between individual behavior and health has been well demonstrated in the literature, elucidating inter-relationships between biological, psychological, and societal influences on individuals' behaviors and, ultimately, health outcomes (1). Many health behaviors are interconnected and tend to occur together. For example, misuse of alcohol, tobacco, and other substances has been associated with multiple concurrent sexual partnerships and lack of condom use during sex (2–5). Interrelationships between emerging behaviors such as excessive screen time and poor sleep habits with physical inactivity have also been demonstrated (6). On the other hand, prompt and appropriate health care-seeking behavior are associated with positive outcomes (7, 8). In this way, health behaviors can work both independently and synergistically to positively or negatively influence health outcomes and chronic diseases such as obesity, coronary heart disease and cancers (9, 10).

The readiness of the U.S. Armed Forces highly depends on the availability of active-duty service members (ADSM) who are physically and mentally fit, healthy, and able to perform at their peak in a variety of austere and demanding environments. The Department of Defense (DOD) Total Force Fitness framework includes eight fitness domains for military readiness and resilience, targeting physical, behavioral and psychological fitness (11). DOD seeks to improve the health status of ADSM by increasing access to healthy foods, promoting opportunities for physical activity, and reducing the prevalence of tobacco smoking, binge drinking and other health behaviors (12, 13). However, recent data show rising rates of obesity, problem drinking and sleep disorders among ADSM (14, 15). Health promotion initiatives such as the comprehensive soldier and family fitness program (16) help ADSM to be psychologically fit and resilient across family, social, spiritual, emotional and physical dimensions. Such programs improve ADSM awareness of high-risk behaviors and provide a holistic prevention training program that teaches long-lasting skills that can help ADSM succeed in all aspects of life, producing benefits in times of conflict and in times of peace.

Improving healthy behaviors among ADSM relies on an in-depth understanding of ADSM's lived experiences and the drivers of their engagement in high-risk behaviors to develop contextually relevant and sustainable solutions (4, 17–22). Few studies have examined the clustering of health behaviors among ADSM for the purpose of designing tailored interventions (23, 24). Audience segmentation, the process of identifying clusters or homogeneous sub-populations, has been used in health promotion to develop marketing, behavior change or communication strategies customized to the characteristics of each sub-group (25). These segmentation strategies typically consider the demographic, socioeconomic, cultural, psychographic characteristics and behavioral patterns of a target audience to identify clusters or subgroups that respond or relate in similar ways (26). This strategy is needed because sub-groups may have unique sociodemographic characteristics, value different benefits associated with a behavior, prioritize considerations differently, seek and obtain information or social support for behavior change through different channels, and respond more readily to some message formats than others (27).

There is scant literature that specifically explores the clustering of health behaviors among ADSM. This understanding is critically needed to inform the design and implementation of tailored social and behavior change interventions and health promotion programs that meet the unique needs of ADSM. To fill these gaps, this study used latent class data analytical methods to investigate health behavior patterns among US ADSM to provide targeted recommendations for relevant programmatic and behavioral interventions. This study investigated the clustering of established (physical inactivity, alcohol drinking, tobacco/substance use, health care-seeking and unsafe sexual practices) and emerging health behaviors (screen time and poor sleep) among US ADSM. Furthermore, the study identified profiles of high-risk behavior and explored their association with ADSM characteristics and mental health status. The study hypothesizes that there would be clusters that reflect the spectrum of risk behaviors ranging from low risk taking to high risk taking.

## 2 Methods

### 2.1 Data source

Data analyzed were from the 2018 Health Related Behaviors Survey (HRBS) – a recurring cross-sectional survey by the Department of Defense (DoD) implemented since 1980 to date and currently sponsored by the Defense Health Agency (DHA). The survey aims to understand the health, health-related behaviors, and wellbeing of service members using validated measures that facilitate comparisons with civilian populations (15). The 2018 HRBS is the most recent iteration of the survey with publicly available data. This study focused on the responses of active-duty servicemembers and excludes the reserve (non-active-duty) servicemembers.

### 2.2 Sampling

The inclusion criteria for the HRBS includes all active component personnel as of September 2018 who were not enrolled as cadets in service academies, senior military colleges, and other Reserve Officers' Training Corps programs. The HRBS sampling frame utilized 50 strata based on the combinations of sex (two categories), service branch (five categories), and pay grade (five categories). From the sampling frame of 1,357,219 servicemembers, 199,996 active-duty service members were randomly selected and approached to complete the online survey. A total of 17,166 surveys were completed with a response rate of 9.6%. The study slightly oversampled women, Marines, and junior enlisted personnel to guarantee enough of those groups to yield reliable estimates. Design weights accounted for this oversampling and non-response weights addressed strata-level differences in response rates to the survey. The final analytic weights were the product of the design and nonresponse weights in order to make the analytic sample representative of the ADSM in the US. In the HRBS 2018 data, the missingness rates ranged from 0.1 to 7% and was addressed by imputation using predictive mean matching to impute binary, ordinal, and continuous variables, whereas polytomous regression was used to impute categorical data (15).

### 2.3 Variables

#### 2.3.1 Health behaviors

To identify profiles of health behaviors among ADSM, we included seven binary (yes vs. no) indicator variables. These indicators were selected per their availability in the HRBS and based on apriori knowledge of their association with physical and mental health status. This was comprised of the following five short-term and two long-term self-reported behaviors within the past 30 days and 12 months, respectively:

1. Infrequent physical activity and/or strength training in the past 30 days defined as < 3 days per week of strength training, moderate or vigorous physical activity.
2. Screen time (spent using a device with a screen for activities other than for work or school) over 2 h/day in the past 30 days.
3. Poor sleeping habits in past 30 days defined as the presence of all the following: (a) sleeping less than seven hours/day, (b) self-rated quality of sleep as insufficient, (c) the use of prescription medications to go to sleep, or the use of energy drinks, caffeinated beverages, or over-the-counter medications to stay awake.
4. Weekly binge drinking of alcohol in the past 30 days.
5. Tobacco smoking or drug (marijuana or hashish, synthetic cannabis, other illegal drugs, inhalants, synthetic stimulants, non-prescription cough or cold medicine to get high) use in the past 30 days.
6. No visit to the doctor for a routine checkup in the past 12 months.
7. Multiple (two or more) sexual partners or sex with a new partner without a condom in the past 12 months.

#### 2.3.2 Mental health status

The HRBS also included a measure of an ADSM's self-reported mental health based on the validated Kessler Mental Health Scale (K6) battery of questions to assess non-specific serious psychological distress in the past 30 days (28). The score ranges between 0 and 24 with “serious distress” being identified as a mean K6 score >12 (15).

#### 2.3.3 Covariates

These included sociodemographic variables identified from the literature or a priori as associated with risk behaviors and health outcomes including mental health status. Covariates included: (i) sex (male or female), (ii) age group (17–24 or ≥25 years) (iii) race/ethnicity (non-Hispanic Black, Hispanic, White and Other), (iv) education level ( ≤ high school vs. ≥college), (v) marital status (married vs. not married), (vi) service branch (Army, Air Force, Navy, Marine Corps, Coast Guard), and (vii) sexual orientation (identifying as lesbian, gay or bisexual vs. not).

### 2.4 Statistical analysis

Data on socio-demographic, health behaviors, and perceived mental health status were presented as weighted percentages and standard errors (SEs). Bivariate associations were tested using weighted Rao-Scott tests. Latent class analysis (LCA) was employed to cluster ADSMs to mutually exclusive classes based on the conditional probabilities of class membership according to the health behaviors. LCA is a statistical procedure used to identify qualitatively different subgroups within populations who often share certain characteristics (29). This methodology accounts for the multidimensionality of health and behavior and has been widely used to uncover patterns of health behavior and their association with health outcomes (30, 31). The number of classes was selected based on the Akaike and Bayesian information criterion indices, the Vuong-Lo-Mendell-Rubin test, and the Bootstrapped Lo-Mendell-Rubin adjusted LRT test. Weighted logistic regression models were used to assess the ADSM characteristics associated with each latent class as well as the relationship between latent class membership and mental health status. Statistical significance was defined as *p* < 0.05 for all statistical tests. Mplus 8.8 (Muthén and Muthén, 1998–2022) and SAS 9.4 (RTI International, Research Triangle Park, NC) were employed to confirm all analyses.

## 3 Results

Weighted percentages of socio-demographic and health behaviors are shown in Table 1. Of the 17,166 ADSMs, 83.3% were male, 37.8% were younger than 25 years of age, and 58.0% were White. The majority had high levels of screen time use (65.0%) and poor sleeping habits (53.9%) in the past month, while 41.7% reported risky sexual behavior in the past year and 29.7% did not have a routine health check up in the past year. Fewer ADSM reported infrequent physical activity/strength training in the past month (19.3%), current smoking or substance use in the past month (18.6%) and heavy alcohol drinking in the past month (9.8%). Of note, the weighted prevalence of past-month serious psychological distress was 16.4% (95% CI: 15.5%-17.4%) among ADSMs.

**Table 1:** Weighted estimates of socio-demographic, mental health status, and health behaviors among ADSM, HRBS 2018.

**Active-duty service member characteristics**	**Frequency**	**Weighted %**	**Standard error**
**Sex**			0.35
Male	11,813	83.3	
Female	5,353	16.7	
**Age in years**			0.68
17–24	3,642	37.8	
≥25	13,524	62.2	
**Race/Ethnicity**			
White	10,666	58.0	0.62
Black	2,226	16.3	0.50
Hispanic	6,868	16.1	0.48
Other	1,747	9.6	0.34
**Education**			0.51
≤ High school	7,990	65.2	
≥College	8,926	34.8	
**Marital status**			0.64
Married	10,776	53.8	
Not married	6,390	46.2	
**Service branch**			
Air force	5,579	24.1	0.40
Army	3,646	34.5	0.67
Marine corps	2,569	13.9	0.39
Navy	3,675	24.4	0.55
Coast guard	1,697	3.2	0.10
**Sexual orientation**			0.27
Lesbian, bisexual or gay	1,236	6.3	
Not lesbian, bisexual or gay	15,930	93.7	
**Health behaviors**			
Infrequent physical activity/strength training in past 30 days	3,775	19.3	0.46
Screen time over than 2 h/day in past 30 days	10,091	65.0	0.57
Poor sleeping habits in past 30 days	8,825	53.9	0.62
Weekly binge drinking of alcohol in past 30 days	1,336	9.8	0.40
Tobacco smoking or drug use in past 30 days	2,302	18.6	0.53
No routine checkup in the past 12 months	4,494	29.7	0.59
Multiple partners or no condom use with new partner in past 12 months	6,391	41.7	0.62
**Number of health behaviors**			
Zero	1,444	6.2	0.24
One or two	9,375	50.8	0.63
Three or more	6,347	43.1	0.63
**Mental health status in Past 30 days**			0.51
Serious psychological distress present	2,336	16.4	
Serious psychological distress absent	14,830	83.6	

LCA model selection demonstrated (Table 2) the best fit with four (vs. three or five) classes of behavior profiles (Number of free parameters: 31; Bayesian Information Criteria: 129417.538; and Entropy: 0.60).

**Table 2:** LCA Model-Fit Statistics, HRBS 2018.

**Number of classes, free parameters**	**AIC**	**BIC**	**Entropy**	**Bootstrapped likelihood ratio test *P*-value**	**Vuong-lo-mendell-rubin test *P*-value**
3, 23	129216.312	129394.578	0.69	< 0.0001 (2 vs. 3)	< 0.0001 (2 vs. 3)
**4, 31**	**129177.266**	**129417.538**	**0.60**	**< 0.001 (3 vs. 4)**	**0.0145 (3 vs. 4)**
5, 38	129158.642	129460.919	0.31	< 0.0001 (4 vs. 5)	0.3441 (4 vs. 5)

AIC, Akaike Information Criteria; BIC, Bayesian Information Criteria.

Bolded model shows final model selected.

**Figure 1:**
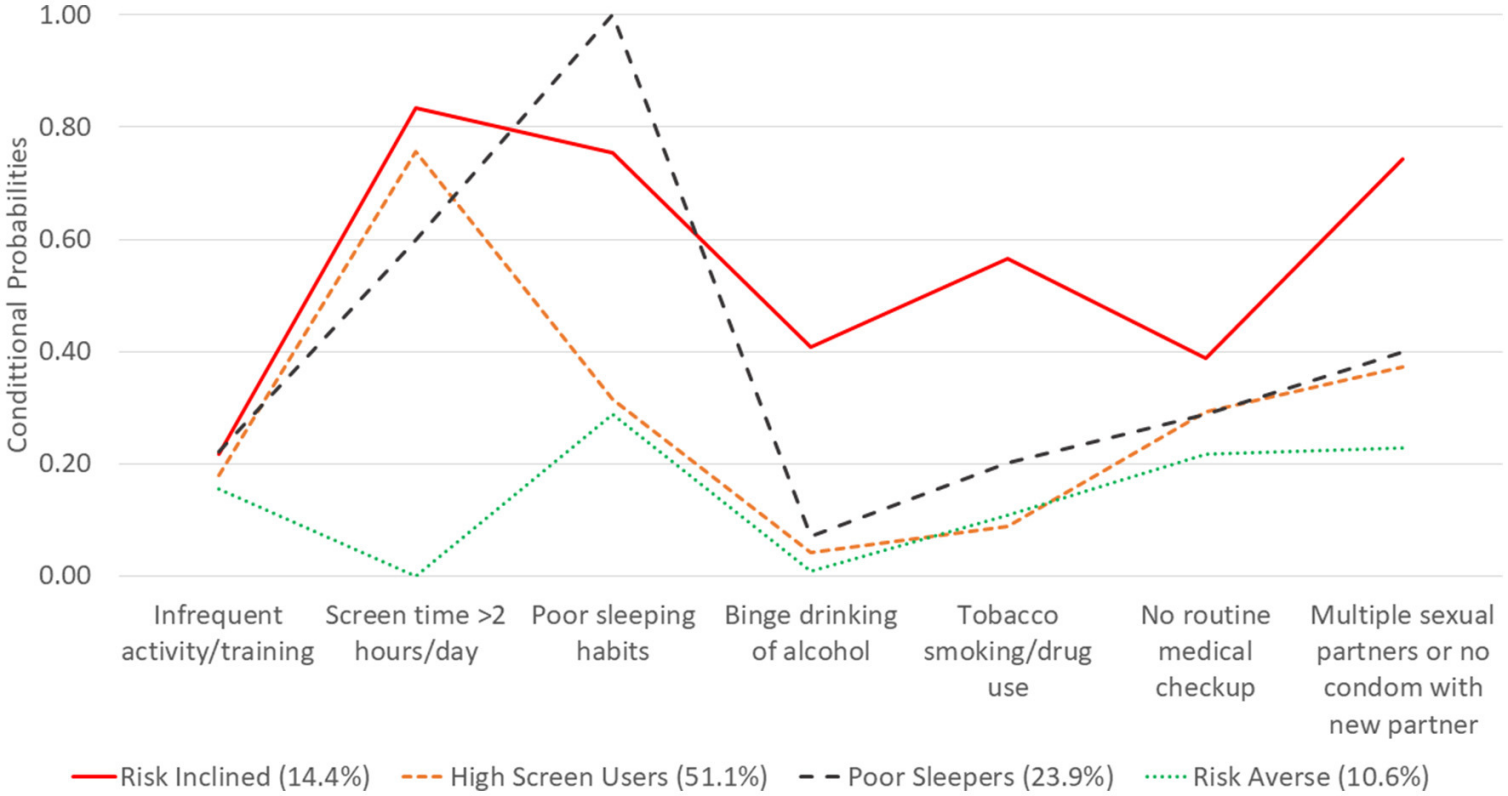
ADSM health behaviors and conditional probabilities of latent class membership, HRBS 2018.

The four distinct classes are described in terms of their risk-behavior profiles in Figure 1. The x-axis lists the names of the behaviors included as indicators in the model. The y-axis provides the conditional probability of class membership for each of the health behaviors. All indicator variables were coded with higher scores reflecting negative behaviors; therefore, probabilities closer to 0 are desirable. The four classes were described as follows based on the distribution of the behaviors. Classes were named if there were any noteworthy (very high or low) behavior or if there was a unique pattern across multiple behaviors.:

1. “Risk Inclined” (14.4% of ADSMs) with higher levels of all behaviors compared to the overall averages and highest levels of high screen time (83.3%), binge drinking (40.9%), tobacco smoking/drug use (56.7%), lack of routine medical checkup (38.9%) and multiple sexual partners or no condom use with new partner (74.3%) compared to other classes.
2. “High Screen Users” (51.1% of ADSMs) with notably highest levels of high screen use (75.6%) compared to other classes.
3. “Poor sleepers” (23.9% of ADSMs) with notably highest levels of poor sleeping habits (100%) compared to other classes; and
4. “Risk Averse” (10.6% of ADSMs) with notably lowest levels of all behaviors compared to other classes.

Table 3 presents the odds of Active-duty Servicemember Behavior Profiles after adjusting for ADSM characteristics (reference categories included female, ≥25 years, white, >high school, not married, Air Force and not lesbian, bisexual or gay). The odds of being described as Risk Inclined (Class I) was significantly higher among ADSM who were males (AOR: 1.78; 95% CI: 1.44–2.20), 15-24 years old (AOR: 1.26, 95% CI: 1.01–1.58), less educated (AOR: 1.90; 95% CI: 1.57–2.31), Army (AOR: 1.49; 95% CI: 1.17–1.91), Marine Corps (AOR: 2.42; 95% CI: 1.95–3.01), Navy (AOR: 2.41; 95% CI: 1.93–3.01), and lesbian, bisexual or gay (AOR: 1.92; 95% CI: 1.45–2.53). On the other hand, ADSM who were Black (AOR: 0.73; 95% CI: 0.55–0.97) or married (AOR: 0.65; 95% CI: 0.52–0.80) were significantly less likely to be described as Risk Inclined.

**Table 3:** Active-duty servicemember characteristics associated with behavior profiles.

**Active-duty Service member characteristics**	**Risk inclined (Class I)**		**High screen users (Class II)**		**Poor sleepers (Class III)**		**Risk averse (Class IV)**	
	**AOR** ^a^	**95% CI**	**AOR** ^a^	**95% CI**	**AOR** ^a^	**95% CI**	**AOR** ^a^	**95% CI**
Male (Reference = female)	1.78	1.44–2.20	0.93	0.84–1.03	0.91	0.81–1.01	0.89	0.75–1.04
15–24 years (Reference = > 25 years)	1.26	1.01–1.58	1.48	1.29–1.69	0.63	0.54–0.73	0.64	0.48–0.85
Black (Reference = white)	0.73	0.55–0.97	1.53	1.32–1.78	0.83	0.71–0.97	0.57	0.45–0.73
Hispanic (Reference = white)	0.89	0.69–1.15	1.28	1.11–1.48	0.90	0.77–1.05	0.67	0.51–0.87
Other (Reference = white)	0.92	0.70–1.20	1.35	1.15–1.58	0.89	0.75–1.05	0.57	0.42–0.76
≤ High school (Reference = no)	1.90	1.57–2.31	0.93	0.85–1.03	1.11	1.00–1.23	0.57	0.48–0.68
Married (Reference = no)	0.65	0.52–0.80	0.82	0.74–0.92	1.30	1.16–1.47	1.58	1.30–1.93
Army (Reference = air force)	1.49	1.17–1.91	0.73	0.65–0.82	1.32	1.16–1.50	0.94	0.78–1.13
Marine corps (Reference = air force)	2.42	1.95–3.01	0.56	0.49–0.65	1.43	1.24–1.64	0.79	0.60–1.04
Navy (Reference = air force)	2.41	1.93–3.01	0.58	0.51–0.65	1.37	1.21–1.56	0.92	0.76–1.11
Coast guard (Reference = air force)	1.17	0.89–1.54	0.71	0.62–0.81	1.20	1.04–1.38	1.50	1.24–1.81
Lesbian, bisexual or gay (Reference = no)	1.92	1.45–2.53	0.86	0.71–1.03	0.98	0.80–1.20	0.61	0.42–0.88

AOR, Adjusted Odds Ratio; CI, Confidence Interval. ^*a*^Adjusted for sex, age group, race/ethnicity, education level, marital status, service branch, and sexual orientation.

ADSM described as High Screen Users (Class II) were significantly more likely to be 15–24 years old (AOR: 1.48, 95% CI: 1.29–1.69), Black (AOR: 1.53; 95% CI: 1.32–1.78), Hispanic (AOR: 1.28; 95% CI: 1.11–1.48) or other race/ethnicities (AOR: 1.35; 95% CI: 1.15–1.58) but significantly less likely to be married (AOR: 0.82; 95% CI: 0.74–0.92), Army (AOR: 0.73; 95% CI: 0.65–0.82), Marine Corps (AOR: 0.56; 95% CI: 0.49–0.65), Navy (AOR: 0.58; 95% CI: 0.51–0.65), or Coast Guard (AOR: 0.71; 95% CI: 0.62–0.81).

ADSM described as Poor Sleepers (Class III) had significantly higher odds of being educated (AOR: 1.11; 95% CI: 1.00–1.23), married (AOR: 1.30; 95% CI: 1.16–1.47), Army (AOR: 1.32; 95% CI: 1.16–1.50), Marine Corps (AOR: 1.43; 95% CI: 1.24–1.64), Navy (AOR: 1.37; 95% CI: 1.21–1.56), or Coast Guard (AOR: 1.20; 95% CI: 1.04–1.38) but less likely to be 15–24 years old (AOR: 0.63; 95% CI: 0.54–0.73) or black (AOR: 0.83; 95% CI: 0.71–0.97).

ADSM described as Risk Averse (Class IV) were significantly more likely to be married (AOR: 1.58; 95% CI: 1.30–1.93) or Coast Guard (AOR: 1.50; 95% CI: 1.24–1.81) but less likely to be black (AOR: 0.57; 95% CI: 0.45–0.73), Hispanic (AOR: 0.67; 95%CI: 0.51–0.87) of other race/ethnicities (AOR: 0.57; 95% CI: 0.42–0.76), less educated (AOR: 0.57; 0.48–0.68) or lesbian, bisexual or gay (AOR: 0.61; 95% CI: 0.42–0.88).

Table 4 shows the relationship between ADSM behavior profiles and the presence of serious psychological distress. Compared to ADSM described as Risk Averse, all other classes were associated with increased odds of serious psychological distress. Specifically, ADSM described as Risk Inclined (Class I) (AOR: 8.30; 95% CI: 5.16–13.36), Poor sleepers (Class II) (AOR: 5.26; 95% CI: 3.38–8.19) and High Screen Users (AOR: 2.44; 95% CI: 1.56–3.82) had the significantly higher odds of serious psychological distress.

**Table 4:** Odds of serious psychological distress by active-duty servicemember behavior profiles.

**Active-duty service member behavior profile [Reference = risk averse (Class IV)]**	**Serious psychological distress**			
	**OR**	**95% CI**	**AOR** ^a^	**95% CI**
Risk inclined (Class I)	11.14	7.00–17.74	8.30	5.16–13.36
High screen users (Class II)	2.87	1.84–4.46	2.44	1.56–3.82
Poor sleepers (Class III)	5.95	3.83–9.26	5.26	3.38–8.19

AOR, Adjusted Odds Ratio; CI, Confidence Interval. ^*a*^Adjusted for sex, age group, race/ethnicity, education level, marital status, service branch, and sexual orientation.

## 4 Discussion

This study sought to explore behavior profiles among ADSM in terms of their physical activity, screen use, sleep habits, alcohol drinking, tobacco/substance use, preventive health care seeking and sexual behaviors. About half of ADSM were described as High Screen Users, about a quarter were described as Poor Sleepers, and about a tenth were described as Risk Inclined and Risk Averse. These profiles slightly align with the hypothesized clusters along the spectrum of risk behavior, ranging from low to high risk taking. Less than a fifth of ADSM self-reported serious psychological distress based on the validated Kessler Mental Health Scale and this was associated with being described as Risk Inclined, High Screen Users and Poor Sleepers. Study findings corroborate existing literature (15) and offer insight into how to operationalize interventions among subgroups of ADSM as these behavior profiles have been found to be associated with sociodemographic characteristics such as age, sex, race/ethnicity, education, marital status and service branch (32).

Study findings highlight health promotion opportunities and potential behavioral and health interventions for each of the distinct risk profiles. ADSM described as Risk Inclined had the highest levels of most risk behaviors including alcohol or tobacco/substance use and lack of routine medical checkup. These ADSM were also more likely to report serious psychological distress. Mental health promotion interventions with this group could focus on the intersections of substance use, mental health, and health care seeking with mental health in order to identify small doable actions to improve wellbeing and reduce high risk behaviors. Given the adverse outcomes related to these risk behaviors, further research should explore how to identify people in this high-risk group during recruitment (33) as other studies suggest that recruitment advertisements for ADSM may target males with high levels of self-confidence, risk taking and emotional stability (34).

The high rates of poor sleep reported by ADSM, particularly those described as Risk Inclined and Poor Sleepers demonstrate that this should be a priority focus for DOD, requiring multifaceted health education and promotion interventions (35). Solutions may include (i) educating ADSM about the signs and impact of sleep deprivation and providing individual strategies to optimize sleep hygiene. e.g., limiting caffeine/stimulant intake or screen use; (ii) instituting duty schedules that ensure 8 h of sleep or provide adequate recovery time as applicable, using well-established techniques like tactical naps and sleep banking; (iii) shifting military cultural norms from a mentality that equates sleep with laziness or weakness to one where sleep is directly linked to service member performance and thus military readiness (36, 37). While the military culture has been predominantly one with powerful group norms, experience and expectations, this culture can be leveraged to positively impact the health behaviors of ADSM (34). Although the influence of leadership support and education were not included in the survey, research suggests that ADSMs who reported that their supervisors supported healthy behavior were more likely to adopt such behaviors (38).

Suboptimal levels of routine medical care seeking among ADSM, particularly those described as Risk Inclined suggests a need to identify and address barriers to accessing and utilizing such services. Health promotion interventions should seek to promote ADSM health literacy, reiterating the accessibility and value of these routine medical checkups. Such interventions should be complemented by supply-side interventions that promote the quality of care, with seamless connections with preventive and treatment services as needed. In addition, interventions could be implemented to foster social norms that support a culture in the military that promotes, rather than stigmatizes or penalizes, health care seeking, particularly for mental and sexual health services.

Study findings can inform the appropriate channels or strategies to be employed in the design of health promotion interventions. The high screen time reported by most ADSM across behavior profiles suggests a high level of digital or technological literacy. Thus, digital health innovations and applications may prove useful in tracking health-related behavior, monitoring potential health risks, and disseminating health communication messages (39). One method could be to use digital health approaches to address sexual and reproductive health among ADSM, given the high rates of sexual risk taking as well as ADSM propensity to use their devices. Alternatively, digital interventions can improve sleep quantity and quality, such as the use of mobile applications to help, prioritize, plan for and track sleep. On the other hand, high rates of screen time are linked with physical effects such as eye strain as well as deleterious psychosocial effects like PTSD and suicide ideation (40). Future research should explore the amount and content of screen time that is appropriate for ADSM and military readiness, weighing the benefits of digital interventions in the current context of high smartphone use and associated social media addiction seen in over 10% of the general US population (41, 42).

Health education and promotion interventions should aim to maintain the low levels of risk behaviors among the Risk Averse group, celebrating healthier decision making and action among ADSM. Furthermore, ADSM with low-risk behavior profiles such as those described as Risk Averse may prove beneficial in the roll-out of interventions as they act as peer-educators or mentors. Supportive policies and interventions, such as the U.S. Army's Comprehensive Soldier and Family Fitness Program, can provide assessment, education, and skills training for ADSM across multiple domains (e.g., physical, emotional, social, spiritual, and family) on an annual basis throughout their career to ensure that ADSM have and keep a low-risk behavior profile (16).

This study reported somewhat high levels of multiple sexual partnerships or lack of condom use with a new sexual partner. Study findings emphasize the importance of promoting healthy sexual behaviors among ADSM (43). Health promotion programs should aim to shift perceptions regarding these risky behaviors, especially among unmarried and less educated ADSM. Structural interventions may include increasing availability of condoms or implementation of regular testing for sexually transmitted infections. Policy interventions may seek to employ a gender equity lens to ensure accessibility to sexual health commodities and services across all service branches (44, 45).

Major study limitations include the reliance on self-reported data typically subject to recall and social desirability bias. The study's cross-sectional study design limits the ability to make causal inferences; and; unmeasured confounding from factors which might influence mental health status. The study only assessed a limited list of behaviors available in the HRBS which worsened health instead of health protection or promotion behaviors. Also, other relevant behaviors such as eating disorders, internet games, betting and gambling disorders, diagnosed psychiatric disorders etc., were not explored. Thus, this study warrants further exploration into underlying pathways and psychological mechanisms for risk behavior profiles demonstrated.

In summary, study findings demonstrate four distinct risk behavior profiles among ADSM and how to target relevant communication, structural and policy interventions to promote healthy behaviors within the US Military, thus improving readiness, resilience and mission success.

## Data availability statement

The data analyzed in this study is subject to the following licenses/restrictions: The data that support the findings of this study are available from RAND and DOD but restrictions apply to the availability of these data, which were used under license for the current study, and so are not publicly available. Data are however available from the authors upon reasonable request and with permission of RAND and DOD. Requests to access these datasets should be directed to anwar.ahmed@usuhs.edu.

## Ethics statement

This secondary data analysis research was reviewed by the Uniformed Services University (USU) Human Research Protections Program (HRPP) which granted ethical approval waiver for the study with a determination of Research Not Involving Human Subjects (Protocol DBS.2022.408). The HRBS study protocol and all methods were carried out in accordance with the Declaration of Helsinki and other relevant guidelines and regulations. Informed consent was obtained from all study participants. The HRBS study were approved by RAND's Institutional Review Board (known as the Human Subjects Protection Committee), the Westat Institutional Review Board, the Coast Guard's Institutional Review Board, the Office of People Analytics, the Office of the Under Secretary of Defense for Personnel and Readiness's Research Regulatory Oversight Office, the Office of the Assistant Secretary of Defense for Health Affairs and the Defense Health Agency's Human Research Protection Office, and the DoD Security Office. All survey materials included the survey report control system license number: DD-HA(BE)2189. The studies were conducted in accordance with the local legislation and institutional requirements. Written informed consent for participation was not required from the participants or the participants' legal guardians/next of kin in accordance with the national legislation and institutional requirements.

## Author contributions

BO: Conceptualization, Formal analysis, Methodology, Supervision, Visualization, Writing – original draft, Writing – review & editing. ZH: Writing – review & editing. PS: Writing – review & editing. OM: Writing – review & editing. AA: Writing – original draft, Writing – review & editing.

## References

[1] Behavioral risk factors among U.S. Air Force active-duty personnel, 1995. MMWR Morb Mortal Wkly Rep. (1998) 47:593–6. 9694640

[2] Anastario MP Tavarez MI Chun H. Sexual risk behavior among military personnel stationed at border-crossing zones in the Dominican Republic. Rev Panam Salud Publica. (2010) 28:361–7. 10.1177/1557988310362097 21308181

[3] Ayer L Ramchand R Karimi G Wong EC. Co-occurring alcohol and mental health problems in the military: prevalence, disparities, and service utilization. Psychol Addict Behav. (2022) 36:419–27. 10.1037/adb0000804 34968085

[4] Bell NS Amoroso PJ Yore MM Smith GS Jones BH. Self-reported risk-taking behaviors and hospitalization for motor vehicle injury among active duty army personnel. Am J Prev Med. (2000) 18:85–95. 10.1016/S0749-3797(99)00168-3 10736544

[5] Bray RM Brown JM Williams J. Trends in binge and heavy drinking, alcohol-related problems, and combat exposure in the U. S military. Subst Use Misuse. (2013) 48:799–810. 10.3109/10826084.2013.796990 23869454

[6] Luxton DD Greenburg D Ryan J Niven A Wheeler G Mysliwiec V. Prevalence and impact of short sleep duration in redeployed OIF soldiers. Sleep. (2011) 34:1189–95. 10.5665/SLEEP.1236 21886356 PMC3157660

[7] Olapeju B Tamene H Ayele M Heliso S Berhanu T Alemayehu G. Psychosocial factors associated with malaria care-seeking in rural Ethiopia. BMC Public Health. (2022) 22:1460. 10.1186/s12889-022-13862-x 35915425 PMC9341112

[8] Olapeju B Bride M Gutman JR Butts JK Malpass A McCartney-Melstad A. Malaria-related psychosocial factors, past antenatal care–seeking behaviors, and future antenatal care–seeking intentions by maternal age in Malawi and Democratic Republic of the Congo. Am J Trop Med Hyg. (2023) 109:277. 10.4269/ajtmh.23-0069 37364859 PMC10397429

[9] Knapik JJ Caldwell JA Steelman RA Trone DW Farina EK Lieberman HR. Short sleep duration is associated with a wide variety of medical conditions among United States military service members. Sleep Med. (2023) 101:283–95. 10.1016/j.sleep.2022.11.015 36470164

[10] Fuehrlein BS Mota N Arias AJ Trevisan LA Kachadourian LK Krystal JH. The burden of alcohol use disorders in US military veterans: results from the national health and resilience in veterans study. Addiction. (2016) 111:1786–94. 10.1111/add.13423 27061707

[11] Bates MJ Fallesen JJ Huey WS Packard Jr GA Ryan DM Burke CS. Total force fitness in units part 1: military demand-resource model. Mil Med. (2013) 178:1164–82. 10.7205/MILMED-D-12-00519 24183762

[12] Troncoso MR Jayne JM Robinson DJ Deuster PA. Targeting nutritional fitness by creating a culture of health in the military. Mil Med. (2021) 186:83–6. 10.1093/milmed/usaa325 33128550

[13] Nindl BC Williams TJ Deuster PA Butler NL Jones BH. Strategies for optimizing military physical readiness and preventing musculoskeletal injuries in the 21st century. US Army Med Dep J. (2013) 5–23. 24146239

[14] Foster SN Brock MS Hansen S Collen JF Walter R O'Connor P. Sleep disorders related to deployment in active duty service members and veterans. Curr Pulmonol Rep. (2016) 5:101–10. 10.1007/s13665-016-0147-7

[15] Meadows SO Engel CC Collins RL Beckman RL Breslau J Bloom EL. 2018 Department of Defense Health Related Behaviors Survey (HRBS). (2021). 10.7249/RR1695 30323988 PMC6183770

[16] McHugh JM. Comprehensive Soldier and Family Fitness Program (Army Directive 2013–07). Washington, DC: US Army (2013).

[17] Anderson MK Grier T Canham-Chervak M Bushman TT Jones BH. Risk factors associated with higher body fat in US Army female soldiers. US Army Med Dep J. (2014) 75–82. 24706247

[18] Bennett AS Golub A Elliott L. A behavioral typology of opioid overdose risk behaviors among recent veterans in New York City. PLoS ONE. (2017) 12:e0179054. 10.1371/journal.pone.0179054 28594892 PMC5464624

[19] Byrne M Deiss R Mesner O Glancey M Ganesan A Okulicz J. Age, race, and at-risk drinking in an HIV-infected U. S military cohort. Mil Med. (2019) 184:e263–e7. 10.1093/milmed/usy318 30690493 PMC6617508

[20] Byrne M Kluz N Sanchez J Berry-Caban C Marcum R Lalani T. Characterizing self-reported sexual risk behaviors and sexually transmitted infections (STIS) among high risk military population who utilize social networking sites to seek sexual partners. Open Forum Infect Dis. (2016) 3:474. 10.1093/ofid/ofw172.337

[21] Dretsch MN Neff D Caserta R Deagle E Hoge CW Adler AB. Rates of behavioral health conditions and health risk behaviors in operators and support personnel in U. S special operations forces. Psychiatry. (2020) 83:358–74. 10.1080/00332747.2020.1768787 32924845

[22] Foran HM Smith Slep AM Heyman RE. Hazardous alcohol use among active duty air force personnel: identifying unique risk and promotive factors. Psychol Addict Behav. (2011) 25:28–40. 10.1037/a0020748 21244121

[23] Pemberton MR Williams J Herman-Stahl M Calvin SL Bradshaw MR Bray RM. Evaluation of two web-based alcohol interventions in the US military. J Stud Alcohol Drugs. (2011) 72:480–9. 10.15288/jsad.2011.72.480 21513685

[24] Malkawi AM Meertens RM Kremers SP Sleddens EF. Dietary, physical activity, and weight management interventions among active-duty military personnel: a systematic review. Mil Med Res. (2018) 5:1–12. 10.1186/s40779-018-0190-5 30591077 PMC6309065

[25] Slater MD. Theory and method in health audience segmentation. J Health Commun. (1996) 1:267–84. 10.1080/108107396128059 10947364

[26] Slater MD Flora JA. Health lifestyles: audience segmentation analysis for public health interventions. Health Educ Q. (1991) 18:221–33. 10.1177/109019819101800207 2055779

[27] Maibach EW. Designing Health Messages: Approaches From Communication Theory and Public Health Practice. California: Sage Publications Inc (1995).

[28] Andrews G Slade T. Interpreting scores on the Kessler psychological distress scale (K10). Aust N Z J Public Health. (2001) 25:494–7. 10.1111/j.1467-842X.2001.tb00310.x 11824981

[29] Weller BE Bowen NK Faubert SJ. Latent class analysis: a guide to best practice. J Black Psychol. (2020) 46:287–311. 10.1177/0095798420930932

[30] Ingledew DK Hardy L Cooper CL. Latent class analysis applied to health behaviours. Pers Individ Dif. (1995) 19:13–20. 10.1016/0191-8869(95)00045-8

[31] Mathur C Stigler M Lust K Laska M. A latent class analysis of weight-related health behaviors among 2- and 4-year college students and associated risk of obesity. Health Educ Behav. (2014) 41:663–72. 10.1177/1090198114537062 24990599 PMC5051694

[32] Hutchinson JW. Evaluating risk-taking behaviors of youth in military families. J Adolesc Health. (2006) 39:927–8. 10.1016/j.jadohealth.2006.05.015 17116529

[33] Albright DL McDaniel J Suntai Z Wallace J. Alcohol misuse among older military veterans: an intersectionality theory perspective. J Addict Dis. (2021) 39:504–12. 10.1080/10550887.2021.1897201 33709881

[34] Knighton R. The psychology of risk and its role in military decision-making. Def Stud. (2004) 4:309–34. 10.1080/1470243042000344786

[35] Foster SN Hansen SL Capener DC Matsangas P Mysliwiec V. Gender differences in sleep disorders in the US military. Sleep Health. (2017) 3:336–41. 10.1016/j.sleh.2017.07.015 28923189

[36] Defense Do. Pentagon Report on Sleep Deprivation and Military Readiness. Department of Defense, Defense (2021).

[37] Lentino CV Purvis DL Murphy KJ Deuster PA. Sleep as a component of the performance triad: the importance of sleep in a military population. US Army Med Dep J. (2013) 98−108. 24146247

[38] Adler AB Bliese PD LoPresti ML McDonald JL Merrill JC. Sleep leadership in the army: a group randomized trial. Sleep Health. (2021) 7:24–30. 10.1016/j.sleh.2020.06.001 32651093

[39] Wienert J Jahnel T Maaß L. What are digital public health interventions? first steps toward a definition and an intervention classification framework. J Med Internet Res. (2022) 24:e31921. 10.2196/31921 35763320 PMC9277526

[40] Perez LG Dong L Beckman R Meadows SO. Movement behaviors associated with mental health among US military service members. Mil Psychol. (2021) 34:211–23. 10.1080/08995605.2021.1987084 PMC10013521 38536360

[41] Busch V Ananda Manders L de Leeuw JRJ. Screen time associated with health behaviors and outcomes in adolescents. Am J Health Behav. (2013) 37:819–30. 10.5993/AJHB.37.6.11 24001631

[42] Cheng C Ebrahimi OV Luk JW. Heterogeneity of prevalence of social media addiction across multiple classification schemes: Latent profile analysis. J Med Internet Res. (2022) 24:e27000. 10.2196/27000 35006084 PMC8787656

[43] Anastario MP Hallum-Montes R Reyes E Manzanero R Chun H. Toward a social theory of sexual risk behavior among men in the armed services: understanding the military occupational habitus. Cult Med Psychiatry. (2013) 37:737–55. 10.1007/s11013-013-9335-x 24101537

[44] Eichler M. Equity in military and veteran research: why it is essential to integrate an intersectional sex and gender lens. J Mil Veteran Fam Health. (2021) 7:143–9. 10.3138/jmvfh-2021-0016

[45] Jeffery DD Beymer MR Mattiko MJ Shell D. Health behavior differences between male and female U. S military personnel by sexual orientation: the importance of disaggregating lesbian, gay, and bisexual groups. Mil Med. (2021) 186:556–64. 10.1093/milmed/usaa539 33306807

